# Linezolid and tedizolid adverse effects: a review on serotonin syndrome, myelosuppression, neuropathies, and lactic acidosis

**DOI:** 10.1017/ash.2024.487

**Published:** 2025-01-27

**Authors:** Adam Greenfield, Erin Deja, Kimberly Lee, Sangeeta Sastry, Barry Rittmann

**Affiliations:** 1 Department of Pharmacy, UPMC St. Margaret, Pittsburgh, PA, USA; 2 Department of Pharmacy, Virginia Commonwealth University Health System, Richmond, VA, USA; 3 Department of Internal Medicine, Division of Infectious Diseases, Virginia Commonwealth University Health System, Richmond, VA, USA

## Abstract

Oxazolidinone antibiotics—linezolid and tedizolid—are often used to treat multidrug-resistant infections. They are highly bioavailable and ideal for transition to enteral therapy when appropriate. However, multiple associated adverse effects are potentially treatment-limiting. The objective of this review is to discuss relevant adverse effects of linezolid and tedizolid, including serotonin syndrome, myelosuppression, neuropathies, and lactic acidosis, and their commonality in real-world experience in the last decade. Mitigation strategies, including the role of therapeutic drug monitoring, are also discussed.

## Introduction

Linezolid and tedizolid are oxazolidinone antibiotics with broad-spectrum gram-positive coverage. They inhibit bacterial protein synthesis by interfering with translation. Linezolid is approved for the treatment of nosocomial and community-acquired pneumonia, skin and skin structure infections, and vancomycin-resistant *Enterococcus faecium* infections, while tedizolid is approved only for the treatment of skin and skin structure infections. They are highly bioavailable in parenteral and enteral formulations making them attractive options for parenteral to oral conversion^1,2^ (Table 1). Recently, off-label use of both drugs for extended durations of therapy is increasingly common (eg, bone and joint infections, nocardiosis, nontuberculous mycobacteria). More frequent reporting of adverse effects (AEs) in real-world cohorts than those reported in initial clinical trials^3^ leads to prescriber hesitancy, unnecessary avoidance of appropriate therapy, and adverse impacts on antimicrobial stewardship efforts. Given linezolid and tedizolid’s unique roles as oral therapeutic options, it is critical to stratify risk factors for AEs, to elucidate proper use. This review serves to characterize the relationship between the oxazolidinones and potential treatment-limiting AEs including serotonin syndrome (SS), myelosuppression, neuropathies, and lactic acidosis. Throughout, we discuss incidence, risk factors, and AE management strategies.

**Table 1.** Linezolid and tedizolid overview

	Dosing (adult)	Dosage form(s)	Indications*	PK	Renal/hepatic dysfunction	Reproductive considerations
Linezolid	600 mg twice daily	Tablet, suspension, injection	Nosocomial, community-acquired pneumonia Skin and skin structure infections Vancomycin-resistant *Enterococcus faecium* infections	Bioavailability: ∼100% Distribution: Vd ∼40–50 L; 31% plasma protein bound Excretion: ∼30% as unchanged drug	Metabolites accumulate in renal impairment. Overexposure possible in hepatic dysfunction No dosage adjustments recommended TDM considered in patients with increased risk for ADEs	May use if benefits outweigh risks Breastfeeding: Present in breast milk (RID %–9%)
Tedizolid	200 mg daily	Tablet, injection	Skin and skin structure infections	Bioavailability: 91% Distribution: Vd 67–80 L; 70%–90% plasma protein bound Excretion: 18% renally eliminated	No dosage adjustments recommended	May use if benefits outweigh risks Breastfeeding: Limited data exist

Note. TDM, therapeutic drug monitoring; ADE, adverse drug event.

*FDA-approved indications.

## Methods

We conducted a literature search using the PubMed database to identify AEs of linezolid and tedizolid. Search terms included “linezolid” or “tedizolid” and “serotonin syndrome,” “toxicity,” “agonist,” “serotonergic agents,” “monoamine oxidase inhibition,” “myelosuppression,” “thrombocytopenia,” “anemia,” “neuropathy,” “peripheral neuropathy,” “optic neuropathy,” “lactic acidosis,” or “metabolic acidosis.” We restricted the review of the literature to the English language, adult patients, and publications released within the previous decade (January 2014–May 2024). The initial search identified 317 studies, all of which were reviewed. Case reports, although reviewed, were largely excluded.

## Linezolid

### Serotonin syndrome

SS is a rare life-threatening condition that occurs from exposure to serotonergic agents, leading to excessive levels of serotonin (5-HT) in the central and peripheral nervous systems. It is often preceded by a new drug exposure or change in dosage of an existing serotonergic medication. Typical presentation is a triad of neuromuscular abnormalities, autonomic hyperactivity, and altered mental status (Table 2).^4^ Diagnosis of SS is challenging as there are no definitive tests of confirmation. Along with validated criteria such as the Sternbach or Hunter criteria, the latter of which is reported to be 84% sensitive and 97% specific,^5,6^ medical toxicologist evaluation remains the gold standard. However, in the absence of these criteria, SS is considered a diagnosis of exclusion. Due to its nonselective, competitive inhibition of monoamine oxidase (MAOI), linezolid can theoretically increase 5-HT levels, causing SS.^7^ This concern limits linezolid’s utility in patients on concomitant serotonergic medications. However, linezolid’s MAOI activity is limited, and few clinical reports link linezolid with SS.^8^ Next, we discuss SS incidence with linezolid monotherapy and combination therapy with psychotropics/opioid analgesics.

**Table 2.** Serotonin syndrome symptomatology

Symptoms	Severity	Examples
Neuromuscular	Mild	Hyperreflexia, intermittent tremor, myoclonus, akathisia
	Moderate	Inducible clonus, ocular clonus
	Severe	Muscular rigidity
Autonomic hyperactivity	Mild	Tachycardia, diaphoresis, dilated pupils
	Moderate	Hyperthermia, hypertension, diarrhea
	Severe	Autonomic instability
Altered mental status	Mild	Anxiety
	Moderate	Agitation, pressured speech
	Severe	Delirium, confusion, seizures, coma
Other	Severe	Acute kidney injury, metabolic acidosis, rhabdomyolysis, DIC

*Adopted from Quinn et al^4^; DIC, disseminated intravascular coagulation.

### Linezolid monotherapy

Limited trials evaluate the effect of linezolid monotherapy and the incidence of SS; however, the risk is low in the absence of additional serotonergic agents. A 4-hospital retrospective cohort study evaluated AEs in 230 patients on linezolid. Sixteen patients received multiple treatment courses within the study period, totaling 248 distinct courses of linezolid, 69% of which were not on any concomitant serotonergic medications. Of 248 AEs identified, there were no cases of SS in patients on linezolid monotherapy.^3^ A 2021 review of the worldwide FDA Adverse Event Reporting System evaluating 11,259 AEs reported for linezolid found no cases of SS in patients taking linezolid without concomitant serotonergic agents.^9^ Finally, a recent systematic review of 84 studies found the composite rate of SS with linezolid monotherapy to be 0.0050%.^10^ Linezolid therapy, when appropriate, should not be avoided due to concerns for the development of SS.

### Psychotropic medications

Two large, retrospective studies reviewed linezolid use in combination with various serotonergic agents. Kufel et al characterized linezolid use with relative doses of concomitant serotonergic agents (low: <33% of maximum daily dose, moderate: 33%–66% of maximum daily dose, and high: >66% of maximum daily dose) and demonstrated an overall incidence of SS of 0.11% (2 of 1,743). Of the 2 patients with possible serotonin toxicity, 1 received linezolid with moderate dose escitalopram and low dose trazodone and the other with moderate dose duloxetine, moderate dose vilazodone, and low dose metoclopramide. Neither patient met Hunter criteria, and only 1 met Sternbach criteria for SS diagnosis. Overall, 67% of patients received at least 1 concurrent serotonergic agent. However, the mean linezolid duration was only 3.8 days, which may underestimate the risk with longer use.^11^ Bai and colleagues conducted a retrospective, population-based observational study (n = 1134) of adults ≥ 66 years prescribed linezolid. Researchers followed patients for 30 days after initiation and assessed for clinically significant SS requiring ambulatory, emergency, or hospitalized care. The incidence of SS was 0.5% (< 6 events). Of the study population, 19% (n = 215) took concomitant antidepressants with a median overlap with linezolid of 7 days (IQR 5–10 d).^12^ Overall, the incidence of SS remains rare in patients receiving linezolid and concurrent psychotropic medications. Linezolid use with a single, concomitant serotonergic agent appears safe; however, SS risk increases when multiple serotonergic agents are concurrently prescribed. Currently, there is insufficient evidence to recommend a clear cutoff for how many serotonergic agents can be given with linezolid.

### Opioid/analgesic medications

Opioids and other analgesics are widely administered for acute and chronic pain. This coupled with the increased use of psychotropic medications prompted the FDA to issue a safety communication^13^ in 2016 regarding the increased risk of SS when used with other serotonergic medications. Mitwally and colleagues conducted a retrospective, observational cohort study to evaluate the incidence of SS in patients receiving concomitant linezolid and opioids. Fentanyl and morphine accounted for 37.7% and 34% of opioid use, respectively. Of 60 patients, only 1 (1.6%) experienced spontaneous myoclonus. However, this developed post-cardiac arrest, making association with SS less likely.^14^ In a case series by Huang et al, the incidence of possible or definitive SS was 1.0% (n = 2) and 0.5% (n = 1), respectively, in a cohort of 194 patients receiving either methadone, buprenorphine, or dextromethamphetamine in combination with linezolid. Most patients received methadone (133 of 194) at less than or equal to 80 mg/day for greater than or equal to 72 hours.^15^ Traver et al noted 2 possible cases of SS among 383 patients in 494 unique encounters in patients receiving methadone (n = 411), buprenorphine (n = 78), or both (n = 5) in combination with linezolid. One case received methadone 10 mg 3 times daily with linezolid for 48.5 days, and the other received methadone 30 mg 3 times daily with linezolid for 8 days. Both patients also received 3 additional serotonergic agents during hospitalization.^16^ Linezolid use appears safe in patients receiving concomitant opioids, including methadone. Like psychotropic medications, serotonergic load should be considered when initiating linezolid therapy.

## Myelosuppression

Myelosuppression is another AE, limiting linezolid use. Thrombocytopenia is most common, though there are reported cases of anemia, leukopenia, and pancytopenia as well. The mechanism is related to reversible, dose-dependent inhibition of mitochondrial protein synthesis.^17^ Linezolid prescribing information reports a 2.4% incidence of thrombocytopenia in a pooled analysis of Phase 3 clinical trials (n = 2046).^1^ However, post-marketing data revealed linezolid-induced thrombocytopenia (LIT) to be much more frequent. A 2023 systematic review and meta-analysis found that 37% of patients across 40 observational studies (n = 6454) experienced LIT with concurrent renal impairment (OR 3.02; 95% CI, 2.32–3.92) identified as the most significant risk factor. Other identified risk factors include age ≥ 65 years, lower BMI, concurrent liver disease, renal replacement therapy, duration ≥ 7 days, and baseline platelet count < 200 × 10^9^/L.^18^ Renal dose adjustments are typically not recommended despite the fact that approximately 30% of linezolid is renally excreted as unchanged drug. Another 50% is renally excreted as inactive metabolites which share chemical features of the parent drug commonly implicated in drug-induced myelosuppression, including an aminophenazone analog structure and an aniline functional group. In patients with CrCl < 30 mL/min, exposure to these metabolites may be up to tenfold higher, likely contributing to incidence of LIT.^1^ Therefore, it is prudent to consider whether renal dose adjustments are warranted.^19^

## Neuropathies

### Optic neuropathy

Linezolid-induced optic neuropathy (LION) is a reversible complication independent of dose and is related to prolonged use of linezolid. LION presents as blurry vision, loss of visual acuity, color vision defect, or photophobia and is thought to be caused by alteration of the mitochondrial oxidative metabolism in the optic nerve.^20,21^ Once identified and linezolid discontinued, improvement in vision typically occurs within 1–3 months.^22^ Routine ophthalmologic screening is proposed at varying intervals for patients receiving prolonged courses of linezolid.^23^ In 3 systematic reviews and meta-analyses of the safety, efficacy, and tolerability of linezolid in patients with multidrug-resistant tuberculosis (MTB), the incidence of LION was 8%–19%.^24–26^ Nunez et al published a systematic review of 33 LION cases from 2002 to 2018. The duration of treatment until symptom onset was ≥ 28 days in 90.6% of cases, and the mean time of exposure to onset of symptoms was 8 and a half months. Most patients received linezolid 600 mg daily. Linezolid was discontinued in all cases with clinical improvement in 31 of 33 patients.^20^

### Peripheral neuropathy

Linezolid-induced peripheral neuropathy (LIPN) is generally irreversible and related to prolonged use and total cumulative dose of linezolid.^27^ LIPN presents as numbness, paresthesia, impairment in pain, temperature, and light touch sensations, or loss of proprioception in a glove and stocking distribution. The mechanism of LIPN is not completely understood but has been theorized to be related to mitochondrial toxicity affecting motor and sensory nerves,^28^ myelin sheath loss, or growth inhibition of Schwann cells.^26,29^ Estimates of LIPN incidence are variable, reported as 6.7%–60% in a systematic review and meta-analysis of 14 studies of linezolid used to treat MTB, with a pooled estimate of 26%.^26^ High incidence rates may be attributed to prolonged durations of therapy for MTB treatment or may be confounded by the concomitant use of other MTB treatments associated with peripheral neuropathy. An open-label study of linezolid use for other chronic infections, including osteomyelitis, device-related infections, septic arthritis, and wound infections, found LIPN incidence to be 12.5% with a median duration of treatment of 80.5 days (range 50–254 d).^30^

## Lactic acidosis

Linezolid-associated lactic acidosis (LALA) is a rare but potentially lethal AE attributed to interference with mitochondrial protein synthesis. LALA presents with metabolic acidosis and elevated serum lactate and is often misdiagnosed. Like SS, LALA reporting has been largely in the post-marketing phase. Multiple studies have sought to identify risk factors associated with the LALA development. Im and colleagues compared 72 patients matched by age and antimicrobial indication who received linezolid to 72 patients who received teicoplanin (control). Definite lactic acidosis, defined as serum pH < 7.25 and serum lactate > 4 mmol/L, occurred in 2 (2.7%) linezolid patients and none of the control patients. Probable lactic acidosis, defined as the occurrence of serum pH < 7.25 or serum lactate > 4 mmol/L, occurred in 3 (4.1%) linezolid patients and 0 control patients. Among patients receiving linezolid, age > 70 years, concomitant diabetes, and eGFR < 50 ml/min/1.73m^2^ did not contribute to increased change in anion gap. Linezolid duration greater than or equal to 6 weeks was the only risk factor associated with increased anion gap events (*P* = .0014). Although renal impairment did not contribute to the incidence of LALA in this study, it may still affect morbidity and mortality.^31^ Mao and colleagues conducted a review of 47 LALA cases published in 35 unique articles. The onset of LALA ranged from 1 to 109 days of therapy. Overall mortality was 25.5%. No mortality difference was noted by gender, age ≥ 65 years, or duration of linezolid therapy prior to LALA onset.^32^ A retrospective study by Dai et al utilized the FDA Adverse Event Reporting system to identify the reporting odds ratio for linezolid and lactic acidosis. Among 6,218 reports, LALA was identified in 275 (4.42%) cases. Most reports (n = 103, 37.45%) included linezolid as the only suspected drug contributing to lactic acidosis.^33^

The mortality rate of LALA remains high in published literature (20%–26%).^31,32^ Treatment involves discontinuation of linezolid and correction of metabolic acidosis, sometimes necessitating renal replacement modalities and hemodynamic support.^31^ Although it is difficult to establish definitive risk factors for LALA development, added caution should be given to patients receiving linezolid for prolonged courses and those with underlying disease states that may predispose to increased morbidity and mortality.

## Prevention of AEs

Many AEs associated with linezolid are concentration dependent, yet no manufacturer recommendation exists for therapeutic drug monitoring (TDM), target serum concentration, or dose adjustments. In a study by Song and colleagues, mean linezolid trough (C_min_) concentrations were inversely correlated with mean mitochondrial function levels and directly correlated with risk for AEs. For every 1 mg/L increase in linezolid C_min_, there was a twofold increase in risk for AEs (HR 2.05; 95% CI, 1.05–2.79).^34^ Significantly, increased risk for myelosuppression has been noted for linezolid C_min_ > 8 mg/L.^35^

Linezolid displays time-dependent antimicrobial activity with an estimated pharmacodynamic target of AUC_24_/MIC > 100. A 2019 pharmacokinetic study estimated that a linezolid C_min_ of 2–8 mg/L for organisms with MIC ≤ 2 would achieve this target in most patients.^36^ In a 10-year retrospective study of linezolid C_min_ in 1049 patients treated with linezolid 600 mg q12h with 2484 unique levels, only 50.8% were within the specified goal range of 2–7 mg/L, and overexposure occurred much more frequently than underexposure (33% vs 16.2%). Overexposure was significantly associated with CrCl ≤ 40 mL/min (OR 1.463; 95% CI, 1.124–1.904).^37^ An expert consensus statement published in August 2022 suggests linezolid TDM to maintain trough concentrations of 2–8 mg/L, particularly in patients who are critically ill, elderly, obese, with decreased or augmented renal function, liver cirrhosis, or taking medications that interact with linezolid. Experts recommend an initial dose of 300 mg q12h for patients with CrCl ≤ 30 mL/min, although this could be adjusted based on clinical considerations and TDM.^38^ A retrospective study published in 2023 evaluated the impact of linezolid TDM with a goal C_min_ of 2–7 mg/L. Of 622 patients included in the study, 144 patients received TDM (23%). The median first concentration was 8.25 mg/L, suggesting overexposure for much of the study population. A multivariable model demonstrated that appropriate dose adjustment based on TDM significantly reduced the odds of linezolid toxicity (aOR = 0.45; 95% CI, 0.21–0.96). The overall incidence of thrombocytopenia and peripheral neuropathy in the study was 9.8% (61 of 622) and 1.9% (12 of 622), respectively. This is lower than other reports and likely reflects the impact of the TDM subgroup who received appropriate dose adjustment.^39^

## Tedizolid

### Serotonin syndrome, myelosuppression, neuropathy, and lactic acidosis

Tedizolid is a second-generation oxazolidinone FDA approved in 2014 for acute bacterial skin and skin structure infections. Similar to linezolid, tedizolid demonstrates reversible monoamine oxidase A and B inhibition, however to a much lower degree *in vitro*.^40^ It is also more potent *in vitro* against gram-positive pathogens, allowing for lower total daily doses. Therefore, the standard dose of tedizolid results in lower free drug plasma concentrations, causing lesser inhibition of mitochondrial protein synthesis. Tedizolid is primarily eliminated via the liver as an inactive sulfate conjugate with limited renal clearance. These pharmacokinetic advantages may account for a decreased propensity for tedizolid to induce neuropathy and myelosuppression.^40,41^

A review of FDA Adverse Event Reporting System data between 2014 and 2020 included 271 tedizolid and 11,259 linezolid reports. There were no significant differences observed between tedizolid and linezolid regarding (0.98, 95% CI, 0.63–1.52), peripheral neuropathy (0.63, 95% CI, 0.28–1.42), SS (0.44, 95% CI, 0.16–1.19) or lactic acidosis (0.61, 95% CI, 0.22–1.64).^8^ A retrospective study of 81 patients treated with tedizolid for a median of 28 days reported only 6 (7.4%) developed thrombocytopenia during treatment. No patients developed peripheral or optic neuropathy, lactic acidosis, or experienced SS, though only 7 patients received concomitant serotonergic agents. Notably, nearly half of the study population had previously received linezolid, and 27.2% experienced linezolid toxicity.^42^ An open-label, non-comparative trial of oral tedizolid for bone and joint infection in 37 patients saw no cases of any cytopenia, new or worsening peripheral or optic neuropathy, or incidence of SS with a median treatment duration of 10.1 weeks.^43^ A case series by Morrisette et al included 37 patients treated with tedizolid for a median of 188 days and found no significant difference in complete blood count indices from baseline to last laboratory values obtained while on tedizolid. Eight (21.6%) patients experienced new onset thrombocytopenia, and no patients experienced severe thrombocytopenia, defined as platelet count < 50 × 10^9^/L. No patients developed new or worsening peripheral neuropathy, optic neuritis, or SS despite 54.1% of patients receiving at least 1 concomitant serotonergic agent, and 27% receiving 2 or more. One patient discontinued tedizolid after developing lactic acidosis, although this occurrence was likely confounded by concomitant medication use.^44^ Overall, the risk for AEs with tedizolid is relatively low and may be an alternative option for patients unable to tolerate prolonged durations of linezolid.

## For antimicrobial stewards

Knowledge of common oxazolidinone AEs and associated risk factors play a significant role in antimicrobial selection and stewardship efforts. Oxazolidinone avoidance in patients at low risk for AEs can expose them to unnecessarily broad antimicrobials and suboptimal therapies. Transitioning from intravenous to oral therapy is a core antimicrobial stewardship activity that leads to improved costs and patient satisfaction with similar outcomes and decrease in catheter-related complications.^45^ Intravenous therapy superiority to oral therapy dogma is changing, and linezolid and tedizolid are well-positioned to provide highly bioavailable, broad-spectrum coverage. We believe this review clarifies treatment-limiting AEs and empowers clinicians and antimicrobial stewards to confidently utilize oxazolidinone antimicrobials when clinically appropriate.

## Conclusion

**Figure 1.**
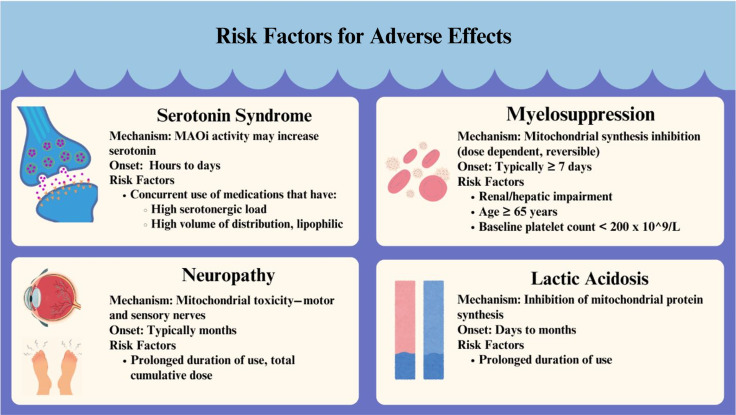
A summary of adverse effects when using linezolid, highlighting mechanism of action, time of onset, and risk factors.

SS with linezolid is exceedingly rare with monotherapy and uncommon in combination with other serotonergic agents. The number, relative dosage, and propensity of each agent to cause SS should be considered when initiating linezolid. The current literature likely underestimates SS rates due to the absence of clear case definitions. Following prompt recognition and discontinuation of linezolid and other offending agents, patients recover rapidly.^10^ Other AEs, such as neuropathies and myelosuppression, may be associated with prolonged courses of therapy and high serum concentrations (Figure 1). These effects are also generally reversible after discontinuation of the drug, except for peripheral neuropathy. Patients should be monitored closely for these effects and TDM considered for patients at high risk for overexposure. The real-world use of tedizolid, although limited, has demonstrated a similar, if not improved, safety profile compared to linezolid. Understanding linezolid and tedizolid adverse events allows for their safe use in clinical practice and can help antimicrobial stewardship efforts to optimize treatment regimens and facilitate intravenous to oral transitions.
